# High throughput DNA extraction of legume root nodules for rhizobial metagenomics

**DOI:** 10.1186/s13568-019-0771-z

**Published:** 2019-04-10

**Authors:** Gabriel Johnson

**Affiliations:** Department of Botany, National Museum of Natural History, Smithsonian Institution, Museum Support Center, 4210 Silver Hill Road, Suitland, MD 20746 USA

**Keywords:** Legume, Root nodule, High-throughput, DNA extraction, Rhizobia

## Abstract

A novel combination of tissue homogenization, cell lysis, and DNA purification techniques was developed for isolating total DNA from whole legume root nodules on an automated robotic system. Silica dehydrated root nodules from soy bean, *Glycine max* cv. Tara, and mung bean, *Vigna radiata* cv. Crystal, were homogenized in 96-well plates and were enzymatically and chemically lysed before being loaded into the AutoGenprep 965 for automated phenol–chloroform based DNA isolation. The resulting DNAs were of relatively high concentration, low fragmentation, and free of phenolic and polysaccharide contaminants. In contrast to manual methods, this adaptation of the Autogen Prep automated DNA extraction instrument allows for 100’s to 1000’s of samples to be prepared in a fraction of the time and cost.

## Introduction

Nitrogen fixation by rhizobial endosymbionts occurs in specialized root structures called nodules and is essential for the integrity of both natural and agronomic ecosystems (Becker 2017; Beijerinck 1888; Hellriegel and Wilfarth 1888). Controlled inoculation of pulses has long been shown to improve agricultural productivity (Brooks 1901; Cotrell et al. 1900; Hopkins 1904). Due to their great importance in crop studies, a diversity of techniques has been developed to allow for the rhizobial endosymbionts to be cultured and isolated for characterization and inoculation studies. While high quality DNA can be purified from certain species that readily culture in vitro, genetically profiling recalcitrant strains requires DNA to be extracted directly from root nodules. Current methods for nodule DNA extraction either result in a crude isolate that can only be used in amplicon-based Sanger sequencing (e.g. Prepman Ultra Sample Preparation) or require expensive manual extraction kits that make large-scale sampling intractable and cost prohibitive.

Since the genera of agriculturally important rhizobia are relatively well known, primers targeting specific variable regions have been developed to profile these strains with amplicon-based methods (e.g. 16S ribosomal DNA, Multilocus Sequence Analysis). This highly developed methodology allows for even minute amounts of poor quality DNA to be analyzed; however, the primers used in these studies cannot be used to explore the vast diversity of nodule rhizobia found in non-crop legumes around the world (Zahran 2001; Weir et al. 2004). Moreover, the high frequency of genetic recombination and horizontal gene transfer among rhizobia and non rhizobial bacteria lead to inaccurate conclusions when based on a small fraction of the genome (Eardly et al. 2005). Although molecular studies of non-agricultural legume root nodules are relatively few (Batzli et al. 1992; Eardly et al. 2017; Freitas et al. 2014; Le Roux et al. 2014; Ma et al. 2012; Sarita et al. 2005), they consistently uncover novel species of nitrogen fixating bacteria that cannot be fully characterized using primers developed for agricultural studies.

Recent metagenomic studies of the legume nodules have shown that they possess a far more diverse array of bacterial species than previously thought (Hartman et al. 2017; Wang et al. 2018). In fact, a single legume nodule may contain populations of many rhizobial and non-rhizobial species aside from the predominant bacterioid symbiont (Hakim et al. 2018; Liu et al. 2007; Wigley et al. 2017; Xiao et al. 2017). Indeed, sequencing only nodule occupants isolated on agar media disproportionately overrepresents those taxa most amenable to in vitro culture. To circumvent this inherent bias, Hakim et al. have demonstrated that direct sequencing of DNA extracted from whole nodules is necessary (2018). These discoveries were made possible by deep sequencing using next-generation sequencing technology and would have been easily overlooked with more traditional methods. However, in order for massively parallel sequencing libraries to be prepared, DNAs of high quality and concentration must be isolated from whole nodule tissue. Such nodule isolations are hampered by the copious amounts of extracellular polysaccharides produced by the symbionts and their host plants in root nodules (Ghosh and Maiti 2016). These mucopolysaccharides are essential for the function of the nitrogen fixating bacteria and have been reported to inhibit many enzymes used in molecular biology (Patrick Elia, personal communication).

As the cost of DNA sequencing continues to decrease exponentially with continual advancements in technology and computing, it is important to have reliable high-throughput DNA isolation procedures to utilize these new technologies at their maximum potential. The AutoGenprep 965 is a robotic liquid handling system capable of performing the phenol chloroform isolation method used for most animal tissues and is a modification of the CTAB chloroform method used with plants (Doyle and Doyle 1987). While this instrument is very effective in isolating high-quality DNA from thousands of samples taken from model organisms (Guttikonda et al. 2014), it has also been used to effectively purify DNA from non-model plants as well (Attigala et al. 2014). In a study of marine snails, the bacterial flora of gill tissues had been genetically profiled using DNAs extracted with the AutoGenprep (Beinart et al. 2012).

The goal of this study was to modify the existing DNA extraction methods used with the AutoGenprep 965 to extract DNA from legume root nodules. The high throughput DNA extraction method described here offers a comparatively inexpensive way to explore the metagenomics of nodule rhizobia on a scale not heretofore accomplished.

## Materials and methods

### Samples

Root nodules were obtained from two species: soybean (*Glycine max* L. cv. Tara) (N = 34) and mung bean (*Vigna radiata* L. R. Wilczek cv. Crystal) (N = 16). These specimens were collected as root masses taken from agricultural fields at the Beltsville Agricultural Research Center (Beltsville, MD, USA by Patrick Elia in the spring of 2017 and soon frozen at − 20 °C. Shortly before this study, the frozen root mass was placed in a polyethylene bag containing calcium sulfate desiccant (W.A. Hammond Drierite Co. Xenia, OH) to completely desiccate the root nodules.

### DNA extraction protocol

The desiccated root nodules were first surface sterilized with 1.0% (v/v) commercial bleach (Chlorox, Oakland, CA). After weighing the nodules, each were placed in the well of a deep welled 96-well Co-Star plate. 200 µl of the bleach dilution was added to each well and the nodules were gently agitated in the solution for 1 min. The bleach solution was removed and replaced with 200 µl of sterilized water. After three water washes, the nodules were transferred to a deep welled 96-well Matrix plate in which the wells had been pre-filled with ~ 50 µl of 0.3 mm sharp garnet particles (BioSpec Products Inc., Bartlesville, OK). After the nodules had been added to the plate, a 5.0 mm stainless steel bead (OPS Diagnostics, Lebanon, NJ) was added to each well. This mixture of beads approximates “lysing matrix A” produced by MP Biomedical (Santa Ana, CA).

Cell lysis buffer solutions were formulated based on Krasova-Wade and Neyra (2007). To hydrate and soften the nodules, 100 µl of TES buffer (20 mmol Tris–HCl, pH 8.0, 50 mmol disodium EDTA, pH 8.0, 50 mmol NaCl, 8% (w/v) sucrose) was added to each well and the plate was incubated at 37 °C for 15 min. After closing the wells with 8-well cap strips and strapping a plexiglass pane over the tops of the plates, the plates of nodules were loaded into the FastPrep96 bead beater and were shaken at maximum speed for 30 s, then repeated to completely homogenize the tissue. To each well, 10 µl of 20 mg/ml lysozyme solution (20 mM TrisHCl, pH 8.0, 2 mM sodium EDTA, 1.2% Triton^®^ X-100, 20 mg/ml (w/v) egg white lysozyme) was added and the enzyme was mixed into the tissue homogenate with manual shaking of the plates. The samples were incubated at 37 °C for 15 min before 250 µl GES buffer (0.5 mmol 1-guanidine thiocyanate, 0.1 mol EDTA disodium pH 8.0, 1% (w/v) *N*-lauroylsarcosine sodium salt) was added to each well and the plates were again manually shaken to mix the solution. The tissue homogenate was incubated with the GES buffer for 15 min at 65 °C with gentle agitation and then was allowed to cool to room temperature.

Cellular debris was cleared from the lysate by centrifugation at 3700×*g* for 20 min at 4 °C. Using the Liquidator 96-channel pipettor, 200 µl of supernatant was transferred into a 96-well Matrix plate in which each well was pre-loaded with 10 µl of 10 mg/ml RNase A (VWR International, Radnor, PA). The cleared lysate was added to the RNase A and mixed by pipetting followed by a 37 °C incubation for 15 min while being agitated gently. The plates of treated lysate were then loaded into the AutoGenprep 965 and total DNA was isolated using the “Mouse Tail/Tissue” setting according to its default parameters. The RNA digestion step was omitted from the instrument program and the resulting DNA pellets were removed from the instrument after the DNA pellets had been washed for a second time. The remaining ethanol residue was removed by incubating the DNA pellets in the dark at room temperature overnight and DNAs were eluted the next day.

To remove any mucopolysaccharides that may have been co-purified with the DNA, 200 µl of 1× Tris–EDTA buffer was added to each pellet and the plates were incubated at 4 °C for 24 h, undisturbed. The pellet was allowed to passively dissolve into the buffer without any mixing, shaking, or agitation. After the incubation, the plates were carefully transported to the Liquidator 96-channel pipettor so as to not disturb the eluted DNA solutions. The pipettor tips were carefully lowered into the eluted DNA solutions so that they were 2–3 mm submerged beneath the surface of the buffer and not contacting the remaining pellet at all. The topmost 50 µl of the solution was aspirated and transferred to a new plate, leaving the remaining buffer and undissolved polysaccharides at the bottom of the plate wells.

### DNA quantification and analysis

The quality of the DNA extracts was determined by electrophoresis; 2 µl of extract was separated in a 1.0% agarose gel made with 1× Sodium Hydroxide-Boric Acid Buffer (Brody and Kern 2004) and stained with Gel Red (Biotium, Fremont, CA). DNAs were quantified with the Qubit fluorometer (ThermoFisher Scientific, Waltham, MA), and absorbance ratios measured with the NanoDrop C1000 (Wilmington, DE). DNA quantification data were analyzed using R (R Core Team 2013).

## Results

After the surface sterilization with sodium hypochlorite, the dehydrated root nodules began absorbing the rinse water, widening in diameter. They became softer and more fragile when touched with plastic pipette tips. This softening hydration continued with the addition of TES buffer and the initial incubation where the larger nodules had absorbed all of the 100 µl volume added. The bead beating with garnet particles and steel ball bearing rendered the tissue into a fine reddish-brown paste and no discernable tissue fragments. Vigorous vortex mixing and manual flicking was required to incorporate the lysozyme enzyme solution into the thick mixture beads and tissue paste. After resuspension and incubation with the GES lysis buffer, the tissue debris was easily pelletized and separated from the supernatant by centrifugation. The larger nodules yielded lower supernatant volumes since their insoluble tissue debris absorbed a greater amount of buffer.

**Fig. 1:**
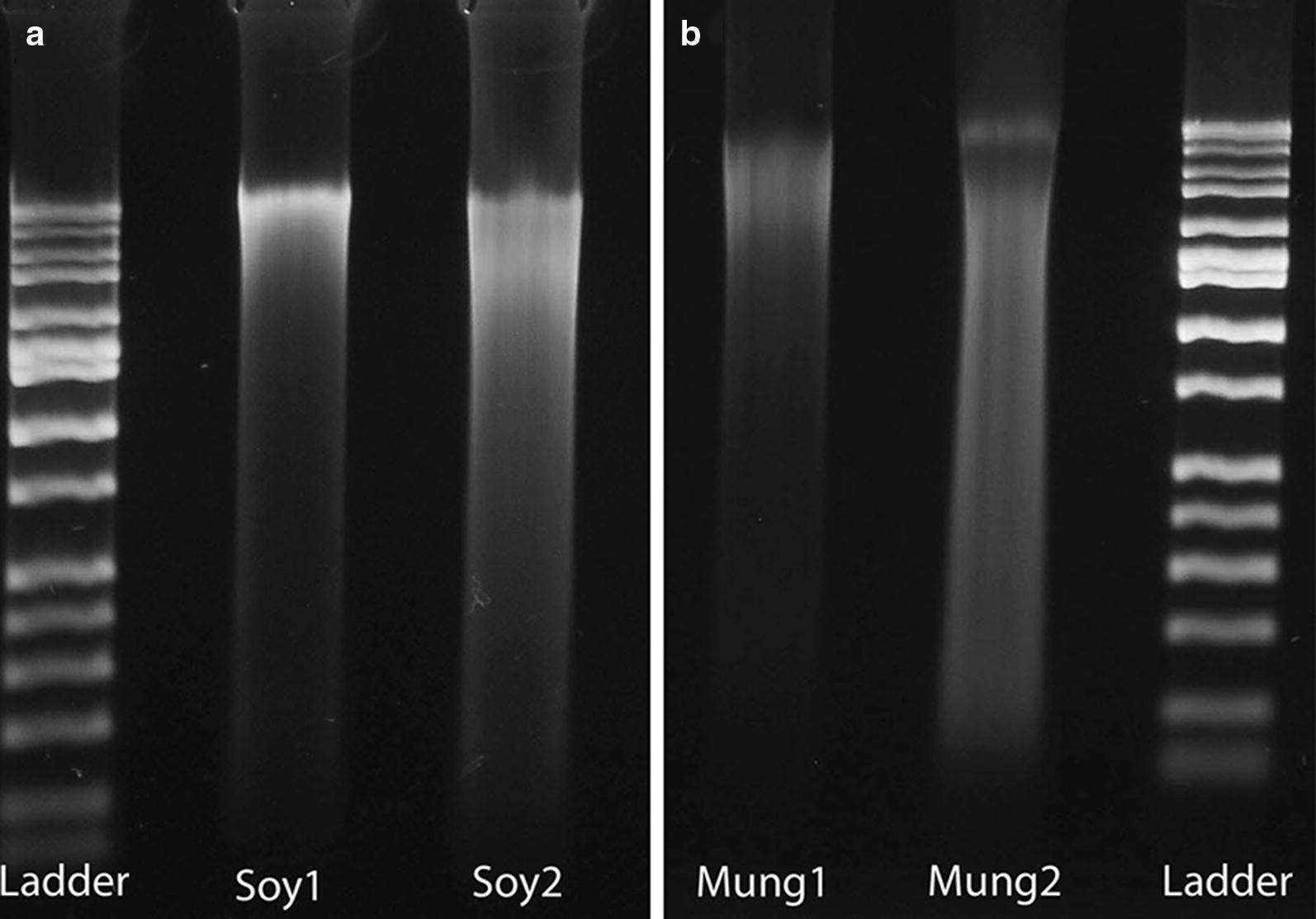
Electrophoretic separations of total DNA isolated from two separate root nodules from *Glycine max* cv. Tara (**a**) and *Vigna radiata* cv. Crystal (**b**) compared to DNA size standard ladders that range from 50 to 10 kb. The concentration and degree of DNA fragmentation varied among samples

The resulting DNAs isolated by the AutoGenprep 965 appeared as tight, round, and slightly translucent pellets at the bottoms of the extraction plates. Upon elution with TE buffer, the extract solutions were either colorless or with a slight tint of pinkish-brown. Spectrophotometric measurements of 260:280 and 260:230 absorbance ratios indicated that the *G. max* nodules yielded a purer DNA extract than those nodules from *V. radiata* (Table 1). Likewise, the mass of DNA obtained per mg nodule dry mass was greater for *G. max* than *V. radiata* as determined with the Qubit dsDNA fluorometric assay (Table 1). Separating these DNA extracts though an electrophoresis gel revealed that a portion of the DNA in each extract had been fragmented, forming a long smear in the gel image (Fig. 1). The presence of brightly fluorescing DNA at or above the top 10 Kb band of the HiLo size ladder indicates that the extracts contained a portion of high molecular weight DNA as well.

**Table 1:** The mean root nodule dry-mass and the respective mean concentration, and 260:280 and 260:230 absorbance ratios of the DNA extracts for *Glycine max* cv. Tara and *Vigna radiata* cv. Crystal

Taxon	Mass (mg)	[DNA] ng/µl	DNA (µg)	DNA/mg	(260:280)	(260:230)
*Glycine max*						
Mean	7.29	144.6	7.23	1.09	1.92	1.48
Median	6.00	74.1	3.70	0.73	1.85	1.53
STD	5.11	150.9	7.55	1.15	0.65	0.71
*Vigna radiata*						
Mean	4.09	18.83	0.94	0.35	1.66	0.94
Median	2.00	12.50	0.62	0.16	1.46	0.80
STD	4.48	19.13	0.96	0.39	0.69	0.47

## Discussion

The mechanical lysis of the cell walls of the plant derived nodule tissue and the bacterial symbionts contained within was achieved through “bead beating” hydrated tissue into a viscous homogenous mass. The degree of DNA fragmentation observed in the final DNA extracts could likely have been reduced by using a less abrasive mixture of grit and beads, such as one similar to “lysing matrix D” manufactured by MP Biomedical and employed successfully by Hakim et al. on mung bean nodules (2018). However, the garnet sharp particles and steel beads used in the present study resulted in a greater consistency of homogenization across samples that varied greatly in density and recalcitrance to mechanical disruption (data not shown).

Initial experiments were conducted with either the CTAB (Doyle and Doyle 1987) and the Phenol–Chloroform–Isoamyl alcohol (Sambrook et al. 1989) default chemistries used by the AutoGenprep 965. The results showed that copious amounts of mucopolysaccharides co-precipitate with the DNA and that the guanidine thiocyanate-based lysis buffer described by Krasova-Wade and Neyra (2007) is essential for reducing this polysaccharide contaminant. Guanidine based lysis buffers also are often the best method for isolating bacterial DNAs (Riffiani et al. 2015) and GES is a key ingredient in most environmental DNA isolation kits for soil. When the guanidine thiocyanate lysis buffer was used with the downstream purification steps of either the Doyle and Doyle (1987) or Sambrook et al. (1989) purification, a greater amount of polysaccharide traces was detected when the Doyle and Doyle method was used (data not shown). Therefore, the DNA extraction method described here is largely a combination of the Krasova-Wade and Neyra (2007) cellular lysis with the subsequent DNA purification steps of the AutoGenprep 965’s phenol–chloroform–isoamyl alcohol chemistry. The use of a guanidine thiocyanate lysis buffer to dissolve mucopolysaccharides was chosen over the use of high salt buffer to preferentially precipitate DNA demonstrated by others (Healey et al. 2014) because such differential precipitation is very sensitive and not amenable to processing on a robotic platform. The final step of differentially dissolving the DNA from an undisturbed pellet was taken from method developed for *Cactaceae* by Martínez-González et al. (2017). In most of the samples, traces of co-purified polysaccharides were not visible in the DNA pellet. This differential solvation step was included in the protocol as an additional precaution that may not be necessary and could be omitted if larger quantities of DNA are needed.

The DNA extraction method described herein successfully isolated DNAs of sufficient concentration and quality for shot-gun Illumina library preparation and with minimal mucopolysaccharide contamination. Although the resulting DNA concentrations varied widely from the mean, even the lowest yields were sufficient for use with nearly all library sequencing kits. This variation in concentrations is evident in electrophoretic gel separations of the DNA extracts; the relative proportions of fragmented and high-molecular weight DNA also varied between nodule samples (Fig. 1). The starting amount of DNA for typical library preparation protocols is 0.1 to 1.0 µg. The average 260:280 nm absorbance ratios were very close to 1.8 suggesting that there was minimal protein contamination (Table 1). The average 260:230 nm absorbance ratio was appreciably lower than the ideal value of 2.0 which is typical of DNA extracted from plants (Drábková et al. 2002; Michiels et al. 2003). This mean 260:230 nm value was even lower for the DNAs extracted from *V. radiata* compared to that for *G. max* perhaps because there was more mucopolysaccharide carry-over in the *V. radiata*. The root nodules of the related *Vigna* species, cow pea, are notorious for producing copious amounts of extracellular polysaccharides that frequently interfere with PCR and other molecular applications (Patrick Elia, personal communication).

Although the average yield of total DNA per mg of nodule tissue was lower than those amounts reported by Krasova-Wade and Neyra (2007) who developed the lysis buffer for manual extraction, the method in the present study is high-throughput and a slight reduction in total yield is a minor inconvenience considering the time and cost savings this method offers.
